# Frontal midline theta rhythm and gamma activity measured by sheet-type wearable EEG device

**DOI:** 10.3389/fnhum.2023.1145282

**Published:** 2023-03-13

**Authors:** Keita Ueno, Ryouhei Ishii, Masaya Ueda, Takuma Yuri, China Shiroma, Masahiro Hata, Yasuo Naito

**Affiliations:** ^1^Department of Occupational Therapy, Graduate School of Rehabilitation Science, Osaka Metropolitan University, Osaka, Japan; ^2^Department of Psychiatry, Osaka University Graduate School of Medicine, Osaka, Japan; ^3^Rehabilitation Unit, Kyoto University Hospital, Kyoto, Japan; ^4^Department of Health Sciences, Graduate School of Health Sciences, Yamagata Prefectural University of Health Sciences, Yamagata, Japan

**Keywords:** EEG, frontal midline theta rhythm (Fmθ), gamma activities, focused attention, calculation, HARU-1, sheet-type EEG device, wearable device

## Abstract

**Introduction:**

The current study measured the frontal midline theta rhythm (Fmθ), which appears in the frontal midline region during the attentional focus state, using the sheet-type wearable electroencephalograph (EEG) device HARU-1, and examined the modulation of frontal gamma band activity by cognitive tasks.

**Methods:**

We measured the frontal EEG of 20 healthy subjects using HARU-1 for 2 min during the rest eyes-closed condition and simple mental calculation task condition, respectively. Statistical analyses were conducted using permutation testing based on *t*-test and cluster analysis to compare the results between the resting state and the task condition.

**Results:**

Twelve of 20 subjects showed Fmθ during the task condition. The 12 subjects with Fmθ showed significantly higher activity of the theta and gamma bands, and significantly low activity of the alpha band during the task condition compared to the resting condition. In the eight subjects without Fmθ were significantly low activity of the alpha and beta bands and no significant activity in the theta and gamma band activity during the task condition compared to the resting condition.

**Discussion:**

These results indicate that it is possible to measure Fmθ using HARU-1. A novel finding was the gamma band activity appearing with Fmθ in the left and right frontal forehead regions, suggesting that it reflects the function of the prefrontal cortex in working memory tasks.

## 1. Introduction

Electroencephalography (EEG) is a leading method for non-invasive measurement of human brain activity and has been used in various fields, from the diagnosis of epilepsy and sleep disorders to brain-computer interface (Teplan, 2002; Padfield et al., 2019). However, EEG amplitude is smaller than electromyography (Raez et al., 2006), electrocardiography (Velayudhan and Peter, 2016), and electro-oculography (Choudhury et al., 2005). Therefore, when measuring EEG, it is necessary to avoid these artifacts. Additionally, power line interference can cause artifacts, limiting the environment. Furthermore, a conventional EEG device has been found to be cumbersome, time-consuming, painful, and uncomfortable to use, requiring skin preparation, gel-electrode application, mounting many wired sensors, and connecting electrodes to the main acquisition unit and personal computer (Mihajlovic et al., 2015).

In the last decade, various types of wearable EEG devices have been developed, and some of them are commercially available. However, most non-clinical EEG solutions are designed for general-use EEG applications with a lack of support for sophisticated signal processing and effective feedback generation, and most of them are used for entertainment such as games (Mihajlovic et al., 2015). In this context, PGV Inc. has released a sheet-type wearable EEG device “HARU-1” (PGV Inc., n.d.a). HARU-1 applies sheet-type EEG electrodes developed by Professor Tsuyoshi Sekitani (Araki et al., 2019). The electrode sheet used in HARU-1 is manufactured by printing electrodes on a flexible and stretchable sheet, which adapts to the movement of the skin surface and does not cause stress or discomfort to the subject (Matsumori et al., 2022). They are low-cost and disposable. The main unit (amplifier, AD converter, wireless signal transmission unit, battery, etc.) is small and light enough to fit in the front forehead. Furthermore, the system is completely wireless, transmitting EEG signals to smartphones and tablets *via* Bluetooth, making it possible to measure EEG during activities without restricting the wearer’s activities (PGV Inc., n.d.a). Li et al. (2019) reported that repeated and longitudinal recording of EEG using sheet-type EEG will contribute to the detection of specific EEG patterns for patients with sleep/wake-related problems to dementia. Matsumori et al. (2022) presented a novel automatic sleep scoring system with HARU-1, and the results indicated that the system is feasible to score the sleep stage with an accuracy comparable to that achieved by clinical polysomnography devices.

One of the characteristic EEG components that appears during mental activity is the frontal midline theta rhythm (Fmθ). Ishihara and Yoshii (1972) discovered Fmθ, which appears in the frontal midline of healthy subjects by using a 13-channel EEG system. Fmθ is induced in an attentional focus, such as during psychological tasks (Ishihara and Yoshii, 1972; Asada et al., 1999; Ishii et al., 2014), and is particularly sensitive to immersion in a task, or when attention is focused on a single task. Fmθ appears in tasks such as the Uchida-Kraepelin test (Ishihara and Yoshii, 1972), rifle shooting preparation (Doppelmayr et al., 2008), and car driving (Laukka et al., 1995), supporting the idea that Fmθ is related to the attentional focus state. Thus, measuring Fmθ during work activities can objectively evaluate the state of attentional focus. In the field of occupational therapy, the appearance of Fmθ during craft activities has been shown to be related to the autonomic nervous responses, suggesting that focusing attention on craft activities can have relaxing effects (Shiraiwa et al., 2020). Also, during cognitive tasks, active mobilization of cortical areas has been reported to be translated into a local increase in the gamma bandwidth (Tallon-Baudry and Bertrand, 1999). Furthermore, the prefrontal cortex (PFC) may influence the posterior sensory areas where working memory representations are maintained (Lara and Wallis, 2015). Using spectral analysis and beamforming, Roux et al. (2012) suggests that gamma oscillations in PFC are critically implicated in the maintenance of relevant working memory information. Previous studies that measured intracerebral EEG during working memory tasks have also reported modulation of gamma-band activity (Mainy et al., 2007; Meltzer et al., 2008). Based on these findings, gamma-band activity may be modulated by cognitive tasks that induce Fmθ.

Since HARU-1 can be used to measure EEG during situations involving movement, it is a suitable device for measuring EEG during work activities and monitoring the subject’s attentional state. However, no attempt has been made to use HARU-1 to measure Fmθ induced by the attentional state, nor gamma-band activity modulated during working memory tasks. This study aimed to validate the utility of HARU-1 for measuring Fmθ and gamma band activity, during a calculation task confirmed to be validated in previous studies.

## 2. Materials and methods

### 2.1. Subjects

The subjects were 20 healthy young volunteers (mean age: 24.6 ± 8.7 years, 8 males and 12 females).

### 2.2. Experimental procedure

**FIGURE 1:**
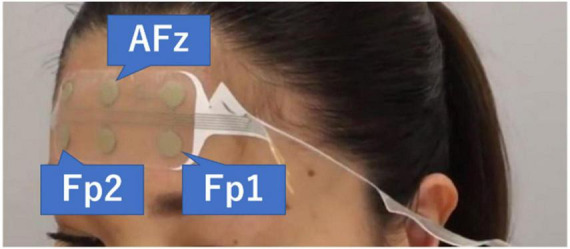
The electrode sheet is attached. The three channels used are AFz, Fp1, and Fp2. The reference electrode was positioned at the left mastoid. PGV Inc. (n.d.b).

Electroencephalography measurements were performed in the sitting position. The room in which the EEG was measured was not a shielded room. However, EEG was measured after confirming that no HAM noise was introduced by real-time spectral analysis using the measurement application HARU-Measure (PGV Inc., Tokyo, Japan). The stretchable electrode sheets (weight = 0.5 g, thickness = 80 μm, stretchability ≤ 150%) used in this study were fabricated by screen-printing an Ag particle-containing paste onto the elastomer substrate that had a moisture permeability of up to 2,700 g m^–2^ day^–1^ (25 μm thickness at 40°C and 90% humidity), which can help avoid bacterial growth even in a wet state (Araki et al., 2019). The reliability of this EEG sensor platform has already been evaluated by comparison with the International 10–20 system, showing a voltage resolution not inferior to that from a high-end device for fixed EEG (Araki et al., 2019). The electrode sheet was attached with a special conductive gel, and the skin of the front forehead and left mastoid region of the subject was wiped with an alcohol swab. The electrode sheet was attached in the same position as AFz, Fp1, and Fp2 based on the International 10–10 system, and the reference electrode was positioned at the left mastoid. The sampling rate was set to 250 Hz (Figure 1).

Electroencephalography signals were transmitted *via* Bluetooth to the measurement tablet LAVIE Tab PC-TE507JAW (NEC Corporation, Japan) and recorded using the measurement application HARU-Measure (PGV Inc., Tokyo, Japan).

**FIGURE 2:**
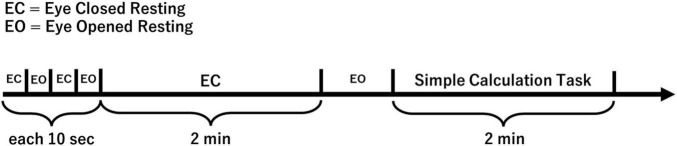
EEG measurement overview.

Below is an overview of the tasks (Figure 2).

Electroencephalography signals were recorded under the following two conditions.

1. Resting eyes-closed condition.

The subjects were instructed to close their eyes while sitting on a chair. The eyes were then kept closed for 2 min.

2. Simple mental calculation task condition.

We adopted a calculation task as an Fmθ-induced task, as in previous studies (Mizuki et al., 1980; Iramina et al., 1996; Ishii et al., 1999). Subjects were instructed to subtract 7 from 1,000 for 2 min in a row with their eyes closed. If subjects calculated while saying the answer or using their fingers, they were instructed to “calculate in your mind.”

### 2.3. Analysis

Electroencephalography data of 20 subjects were visually checked and evaluated whether Fmθ appeared or not by certificated medical doctor of the Japanese Society of Clinical Neurophysiology. The measured EEG data for the resting eyes-closed condition and the simple mental calculation task condition were analyzed using the Brain Electrical Source Analysis: BESA Research 6.0 (BESA GmbH, Germany) software. High Pass Filter of 4.0 Hz, Low Pass Filter of 75.0 Hz, and Notch Filter of 60.0 Hz were applied offline. Two minutes of EEG data in each of the two conditions were divided into 2 s. Artifacts such as blinks and electromyograms were visually removed. Additionally, artifact scans were performed with an amplitude threshold that accepted at least 85% of the remaining epochs. The amplitude thresholds for the two conditions were the same and were set for each subject. The mean value of the amplitude threshold was 102 ± 27 μV. The average number of remaining epochs in the resting eyes-closed condition was 54 ± 6, and the average number of remaining epochs in the simple mental calculation task condition was 50 ± 6. We performed time-frequency analysis in the frequency range of 4.0–75.0 Hz in 0.25 Hz-steps with a time sampling rate of 200 ms-steps by applying complex demodulation to transform time-domain EEG data into time-frequency data which was implemented in BESA (Hoechstetter et al., 2004). A *post hoc* power analysis was performed using G power 3.1 software with a medium effect size (0.5) and α of 0.05 (two-tailed paired *t*-test).

The two groups were set up by certificated medical doctor of the Japanese Society of Clinical Neurophysiology as follows: the Fmθ group (Subjects with theta waves for more than 1 s during simple mental calculation task) and the No-Fmθ group (except for the Fmθ group). Statistical analyses were conducted using BESA Statistics 1.0 (BESA GmbH, Germany) for permutation testing based on *t*-test and cluster analysis (Bornfleth et al., 2020). For the BESA Statistics analysis procedure, we first performed a preliminary test, the paired *t*-test, to define data clusters showing significant effects. After summing all the t-values for each cluster to derive the cluster value, we performed a permutation test based on the cluster value. This method addressed the issue of multiple comparisons. We set “Average over Time” to average the 2 s windows and investigated the statistical difference between the resting eyes-closed condition and the simple mental calculation task condition in each of the two groups. The significance level was set at 0.05.

## 3. Results

### 3.1. Subject classification based on the appearance of Fmθ

**FIGURE 3:**
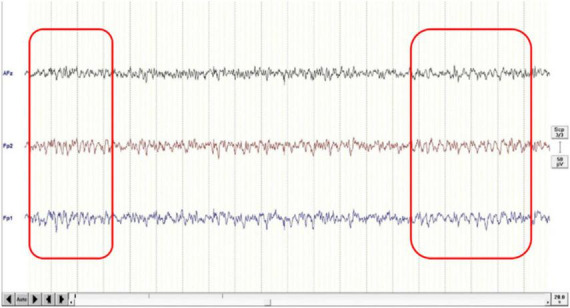
An example of Fmθ lasting more than 1 s during a simple calculation task.

Twelve of the 20 subjects (mean age: 21.2 ± 1.2 years, 4 males, 8 females) showed Fmθ in the simple mental calculation task condition and were assigned to the Fmθ group (Figure 3). The remaining eight subjects (mean age: 29.6 ± 12.0 years, 4 males, 4 females), who did not show clear theta waves in the simple calculation task condition, were assigned to the No-Fmθ group.

### 3.2. Fmθ group

The Fmθ group showed significantly higher activity in theta band in all channels, beta band in all channels and gamma band in Fp1 and Fp2 and significantly lower activity in alpha band in AFz during the task condition compared to the resting condition (Table 1).

**TABLE 1:** Results of permutation test in Fmθ group.

Channel	Cluster value	Mean for task	Mean for rest	Start frequency (Hz)	End frequency (Hz)	*p*-value
Fp1	32.12	4.18	3.15	4.0	7.0	*p* < 0.01
	4.97	2.90	2.52	13.0	13.3	*p* < 0.05
	5.20	1.30	1.20	23.8	24.1	*p* < 0.05
	7.00	1.24	1.14	24.7	25.3	*p* < 0.05
	4.99	1.08	1.01	28.0	28.3	*p* < 0.05
	8.61	0.99	0.89	35.5	36.1	*p* < 0.02
	65.44	0.90	0.79	38.8	45.1	*p* < 0.01
	16.43	0.84	0.74	45.7	47.2	*p* < 0.01
	7.61	0.81	0.73	48.4	49.0	*p* < 0.02
	249.20	0.65	0.57	50.2	73.3	*p* < 0.01
	13.90	0.57	0.49	73.9	75.1	*p* < 0.01
Fp2	16.31	4.13	3.22	4.6	6.1	*p* < 0.01
	5.08	2.98	2.63	13.0	13.3	*p* < 0.05
	12.55	1.37	1.22	24.4	25.3	*p* < 0.01
	17.91	1.20	1.06	27.7	29.5	*p* < 0.01
	28.46	1.15	0.99	30.1	33.1	*p* < 0.01
	19.57	1.10	0.95	34.3	36.1	*p* < 0.01
	400.89	0.79	0.66	39.1	75.1	*p* < 0.01
AFz	4.73	4.37	3.54	4.0	4.3	*p* < 0.05
	8.16	3.14	2.82	5.2	5.8	*p* < 0.02
	-15.53	3.59	4.39	10.3	11.5	*p* < 0.03
	8.01	1.25	1.16	24.4	25.0	*p* < 0.02

Frequency bands with positive cluster values indicate high activity in the task condition, and frequency bands with negative values indicate low activity in the task condition.

### 3.3. No-Fmθ group

The No-Fmθ group showed significantly low activity in theta band in Fp2 and AFz, alpha band in Fp2 and AFz, beta band in all channels and low-gamma band in AFz during the task condition compared to the resting condition (Table 2).

**TABLE 2:** Results of permutation test in No-Fmθ group.

Channel	Cluster value	Mean for task	Mean for rest	Start frequency (Hz)	End frequency (Hz)	*p*-value
Fp1	−33.99	1.28	1.50	19.3	22.3	*p* < 0.01
	−17.45	1.17	1.38	22.9	24.4	*p* < 0.01
	−21.25	1.07	1.24	27.1	29.2	*p* < 0.01
Fp2	−19.17	2.09	2.56	7.6	9.4	*p* < 0.01
	−24.16	1.23	1.43	16.6	19.0	*p* < 0.01
	−28.30	1.34	1.58	19.6	22.0	*p* < 0.01
	−16.83	1.15	1.42	23.5	24.7	*p* < 0.01
AFz	−15.32	2.04	2.77	8.5	9.7	*p* < 0.01
	−23.93	1.19	1.38	16.6	18.7	*p* < 0.01
	−24.66	1.26	1.48	19.3	21.4	*p* < 0.01
	−22.11	1.19	1.39	22.6	24.4	*p* < 0.01
	−11.67	0.87	0.99	32.8	33.7	*p* < 0.05
	−18.21	0.77	0.88	34.3	36.1	*p* < 0.01

## 4. Discussion

In this study, we tested the feasibility of measuring the Fmθ using the sheet-type wearable EEG device “HARU-1.” We found that 12 of 20 subjects (60.0%) showed Fmθ for more than 1 s in the simple calculation task condition. Additionally, the activity of the gamma band was different between the Fmθ group and the No-Fmθ group.

### 4.1. The feasibility of measuring Fmθ using “HARU-1”

In a previous study by Ishihara, 14 university students were asked to perform the Uchida-Kraepelin test, intelligence test, mental calculation, and Tetris, and EEG measurements during the tasks showed that Fmθ was recorded in 5 (35.7%), and long-lasting Fmθ was recorded from 2 of them (Ishihara, 2020). Ishii et al. (2014) performed magnetoencephalography (MEG) recordings using a helmeted whole-head array of 64-channel SQUID sensors and reported that prominent Fmθ was observed in 8 of 11 subjects (72.7%) during the calculation task. Ishihara reported that the Fmθ elicitation rate varies greatly depending on the subject and the eliciting medium and is likely to vary depending on differences in attitude toward the task, the environment in which the subject is placed, and the subject’s personality (Ishihara, 2020). Mizuki et al. (1984) investigated the relationship between the occurrence of Fmθ and personality and reported that subjects who were more anxious, more introverted, and had higher neurotic tendencies showed less or no Fmθ. Tani also reported that increasing motivation for work activities increased the occurrence of Fmθ (Tani, 1978). The subjects who did not exhibit Fmθ in this study were considered to have been unable to focus attention although they were able to perform the task due to the experimental environment, the type and difficulty of the task, and their personalities. In this study, Fmθ during the calculation task was observed in 12 of 20 subjects (60.0%). Our finding is similar to previous studies (Ishii et al., 2014; Ishihara, 2020). This suggests that HARU-1 can measure Fmθ and serve as an objective measure of attentional focus during activities.

### 4.2. EEG characteristics during calculation tasks and attentional focus

In this study, the Fmθ group showed low activity during the calculation task in the α band of AFz, and the No-Fmθ group showed low activity in the α and β bands in all channels. There is a difference in the spectral parameters of the EEG between resting and cognitive tasks, as well as during different mental tasks (Fernández et al., 1995). In particular, the decrease in alpha band activity during mental tasks has been a common observation since the EEG was firstly recorded (Compston, 2010). These previous studies support a significantly low activity of alpha band in all subjects in this study.

We found the gamma band activity only in the Fmθ group during the calculation task. The calculation task used in this study was simple continuous subtraction of 7 from 1,000 which involved several cognitive functions, such as focused attention, executive function, figure manipulation and working memory. Previous studies have reported that the PFC plays a role in maintenance of information, representation, attention, and inhibition in working memory tasks (Miller and Cohen, 2001; Curtis and D’Esposito, 2003). Kaiser and Lutzenberger (2005) reported that synchronized gamma band activity is involved in bottom-up driven perception and top-down driven functions such as selective attention and retention of information in memory. Using spectral analysis and beamforming, Roux et al. (2012) suggests that gamma oscillations in PFC are critically implicated in the maintenance of relevant working memory information. The frontal gamma band activity identified in this study may represent representational maintenance in working memory tasks.

Ishii et al. (2014) and Fitzgibbon et al. (2004) also reported gamma band activity that appears with Fmθ during calculation tasks. Both reports showed increased gamma band activity in the parietal or occipital regions, not sustained frontal gamma band activity associated with Fmθ. The coupling of theta and gamma oscillations has been reported to be associated with the formation of a neural code, recall of sequences of items from long-term memory, and encoding of short-term memory (Lisman and Buzsáki, 2008). Kaplan et al. (2014) suggest that theta phase coupling between medial PFC (mPFC) and medial temporal lobe and theta-gamma phase-amplitude coupling between mPFC and neocortical regions may play a role in human spatial memory retrieval. Therefore, the high gamma band power during the computational task is related to Fmθ and may be theta-gamma coupling that reflects the process of memory processing and recall. As future application, the gamma band activity in the left and right frontal regions observed at the time of Fmθ appearance needs to be verified by multichannel EEG and MEG.

The wearable EEG device is suitable as a tool for assessing and monitoring brain activity in actual daily life because it has few restrictions on activity. The results of this study indicate the possibility that HARU-1 can monitor the attentional state in activities of daily living (ADL). It can aid in the assessment and intervention of difficult ADL in patients with stroke, dementia, and subjects with attention-deficit hyperactivity disorder (ADHD).

### 4.3. Limitations of this study

A limitation of this study was the small number of subjects. The power of the Fmθ group analysis (*n* = 12) was 0.35 and the power of the No-Fmθ group analysis (*n* = 8) was 0.23. Statistical power may not be sufficient, a concept closely related to Type II error. The band of no significant differences between the resting condition and the task condition should be interpreted with caution. Additionally, since this study was conducted on young healthy subjects, it was impossible to examine the effects on the elderly and children, nor was it possible to examine the effects of diseases. And because of many artifacts in the low frequency range and setting the High Pass Filter at 4.0 Hz, frequency bands below the theta band could not be considered.

The tasks that tend to induce Fmθ are those with simple structures, single trials, and appropriate difficulty levels (Ishihara, 2020). Therefore, it is necessary to be careful in interpreting Fmθ when using it to monitor tasks that require the distribution of attention or require multiple trials.

## 5. Conclusion

The HARU-1 can be used to measure the Fmθ induced by the attentional focus state. As a novel finding, we found gamma band activity in the left and right frontal forehead regions that appeared with Fmθ. Future studies are desired that investigate the EEG activity in daily life with more subjects and examine the usefulness of wearable EEG devices in ADL.

## Data availability statement

The original contributions presented in this study are included in the article, further inquiries can be directed to the corresponding author.

## Ethics statement

The studies involving human participants were reviewed and approved by the Osaka Prefecture University Graduate School General Rehabilitation Studies Ethics Committee (2019–203). The patients/participants provided their written informed consent to participate in this study.

## Author contributions

KU, RI, MU, CS, and YN contributed to the design, implementation of the research, and analysis of this study. KU wrote the manuscript with support from RI, MU, TY, and YN. MH instructed KU on how to use the sheet-type wearable EEG device “HARU-1”. All authors contributed to the article and approved the submitted version.
